# Factors associated with free adult preventive health care utilization among physically disabled people in Taiwan: nationwide population-based study

**DOI:** 10.1186/s12913-014-0610-5

**Published:** 2014-12-05

**Authors:** Suh-May Yen, Pei-Tseng Kung, Wen-Chen Tsai

**Affiliations:** Department of Health Services Administration, China Medical University, No. 91 Hsueh-Shih Road, Taichung, 40402 Taiwan; Department of Public Health, China Medical University, Taichung, Taiwan; Department of Chinese Medicine, Nantou Hospita, Nantou, Taiwan; Department of Healthcare Administration, Asia University, Taichung, Taiwan

**Keywords:** Physical disability, Disability, Adult health examination, Preventive health care

## Abstract

**Background:**

Few previous studies have specifically addressed the health care utilization situation of the physically disabled. This study aimed to investigate the utilization of free adult preventive health care for physically disabled people and its’ affecting factors.

**Methods:**

The data was obtained from three nationwide databases from 2006 to 2008. This study comprised 329,264 physically disabled people in Taiwan above the age of 40 who had eligible health checks during 2008. We employed descriptive statistics to analyze the use and rate of free preventive health care use by physically disabled adults. Logistic regression analysis was used to explore the factors that affect physically disabled adults’ use of free adult preventive health care.

**Results:**

16.37% of the physically disabled adults used free adult preventive health care. Women (17.66%), married (17.16%), a junior high education level (17.89%), and mildly disabled adults (18.77%) had the highest use rate among various participant subgroups. The variables that significantly influenced the use of free adult preventive health care by the physically disabled included gender, age, education, marital status, urbanization of the residence areas, monthly payroll, aboriginal status, catastrophic illnesses status, relevant chronic diseases, and severity of disability.

**Conclusions:**

Physically disabled using preventive health care tend to be low. Governments should use the media to reinforce propagation and education of these services to specific, low-utilization groups, and encourage doctors to actively provide preventive health care to communities.

**Electronic supplementary material:**

The online version of this article (doi:10.1186/s12913-014-0610-5) contains supplementary material, which is available to authorized users.

## Background

Because nerve damage from an injury in physically disabled people causes chronic pain [1,2], depression [3], and other complications, these patients lack the motivation to participate in various activities. Physically disabled people often have a sedentary lifestyle, leading to become overweight and obese [4-6]. Being overweight and obese cause changes in the modes of carbohydrate metabolism, increasing the prevalence of diabetes, hyperlipidemia, hypertension, and cardiovascular disease [7,8]. A 2002 study showed that people with disabilities in their lower extremities are 2.5 times the overweight and obesity ratio of average persons, and 40% of people with chronic spinal cord injuries are overweight or obese [9].

Nosek et al. [10] found that physically disabled women had 4 times the likelihood of suffering from diabetes, and 2 times the likelihood of suffering from hypertension than do average women. Studies have shown that those with spinal cord injuries have a higher prevalence of diabetes and coronary heart disease [11-13]. Poliomyelitis patients have a higher hyperlipidemia occurrence than the average person, and typically have two or more coronary artery disease risk factors [14]. Until the end of 2011, Taiwan’s physically disabled population (386,217 people) accounted for 1.66% of the total population, and constituted the group with the most people (35.09%) [15,16] with mentally or physically disabilities (handicapped).

The disabled population has increased annually, consuming the government’s health care and long-term care resources and producing a significant financial burden and challenge for society [17-20]. Maciosek et al. [21] believed that if the government expanded the promotion of free preventive health care utilization for all population, it would save US $3.7 billion annually in medical care expenditures. Increasing the utilization of free preventive health care can delay disease progression, reduce the severity of the disease, and effectively reduce medical expenses derived from complications [22-25]. Eliminating the inequities of disabled persons in health and medical care is the goal of the US *Healthy People* for 2020 [26].

Besides the cancer screening, Taiwan Health Promotion Administration provides at least six free preventive health care programs, including mammography, pap smear test, prenatal examination for pregnant women, children preventive health services, adult preventive health services, and children dental examination with fluoride varnish. The adult free preventive health care includes physical examinations, health education, and blood and urine tests. Adults aged 40 and above are legible this free service, but different age groups have varying frequency limits.

Because of the low mobility of physically disabled people, their medical care time is lengthened. Difficulties in receiving free preventive health care among the disabled people would lead to delay in receiving appropriate medical care. Previous studies showed gender, marital status [16], educational level, age, income, health status, severity of disability, and urbanization level would influence the utilization of preventive health services for the disabled people [27]. Much of the literature related to the disabled people focused on the secondary conditions of the disability, leaving a scarcity of research on the preventive health care needs of this disabled population. Few previous studies have specifically addressed the health care utilization situation of the physically disabled. This study used physically disabled people as participants to explore their free adult preventive health care utilization and its related factors, and to serve as a reference for adjusting preventive health policies for disadvantaged groups.

## Methods

### Data source and participants

The premise for the use of free adult preventive health care includes (1) people suffering from poliomyelitis and older than 35 years (use once a year), (2) people aged between 40 to 64 years (once every three years), and (3) people aged 65 or older (once a year). The data were obtained from three national databases, and all three national databases were managed by the government. The Disabled People Registry File (2008) obtained from the Ministry of the Interior, the free preventive health care file (2006 to 2008) provided from the Health Promotion Administration and the National Health Insurance Research Database (2006 to 2008) released by the Ministry of Health and Welfare.

This study comprised 329,264 physically disabled people in Taiwan above the age of 40 who had eligible health checks during 2008. Among these, 153,117 were mildly disabled, 128,201 were moderately disabled, 44,097 were severely disabled, and 3,849 were extremely severely disabled. Among the 35 to 39 year-old disabled adults, since there was no way to distinguish who were poliomyelitis persons in the Disabled People Registry File and we could not analyze the utilization rate and likelihood of using preventive health care, this group was excluded when the analyses were conducted.

In the present study, demographic characteristics and severity of disability were obtained from the Disabled People Registry File (2008). Socioeconomic status, health status, and the environmental factor were merged from the National Health Insurance Research Database, which is publicly available. The information of using the free adult preventive health care among the physically disabled people was identified from the free preventive health care file (2006 to 2008).

After we applied and were approved to use these three databases, the personal identifications including ID number and name were used to exactly match all people’s data or information in these three databases in the Statistics Center of the Ministry of Health and Welfare, Taiwan. All personal information could be completely linked among these three national databases. All individual’s identification information has been deleted and personal privacy was protected in using these data. This study was approved by the research ethics committee of China Medical University and Hospital (IRB No. CMU-REC-101-012).

### Variables description

The variables in this study were (1) demographic characteristics (i.e., gender, age, marital status, education, aboriginal status); (2) socioeconomic status (i.e., monthly insured payroll); (3) health status (i.e., catastrophic illness/injury, type of chronic illnesses including 15 comprehensive categories of chronic diseases such as cancer, endocrine and metabolic disease, mental illness, disease of nervous system, disease of circulatory system, disease of respiratory system, disease of digestive system, disease of urinary system, disease of skeletal and muscular system and connective tissue, disease of eyes and auxiliary organs, infectious disease, congenital malformation, skin and subcutaneous tissue disorders, disease of blood and blood-forming organs, and disease of ear and mastoid process), severity of disability (i.e., very severe, severe, moderate, and mild); and (4) environmental factors (i.e., urbanization level of residence area, URA; eight levels: Level 1 being the highest urbanized areas, and Level 8 being the lowest). The dependent variable that whether the physically disabled persons used the adult preventive health care was identified in the year 2008 for those aged 65 or older(once per year) and identified in the period of 2006- 2008 for those aged between 40 and 64 (once every three years). The other relevant independent variables, including demographic, socioeconomic, health status, severity of disability, and environmental factors were obtained in the year 2008 from three national databases. The chronic disease groups used in this study were based on “the Range of Chronic Diseases” in the National Health Insurance Research Database. The chronic diseases listed in the Range of Chronic Diseases were defined by the Bureau of National Health Insurance in Taiwan, which consisted of 16 categories of chronic diseases. (Additional file 1). Since the “others” category had very few patients in the disabled people, this study excluded the “others” category in our analysis.

### Statistical analysis

This study used descriptive statistics analysis, chi-squared test, and multiple logistic regression analysis to explain the relationship between the variables. The first step used descriptive statistics to analyze the physically disabled people’s free adult preventive health care utilization quantity and ratio, focusing on their demographic characteristics, socioeconomic status, health status, environmental factors, and other variables. The second step used the chi-square test to compare the difference in physically disabled people’s use of free preventive health care. Since all variables had a p value <0.05 in the chi-squared test in Table 1, we placed all variables into the logistic regression analysis to explore the factors that affected the use of free adult preventive health care among the physically disabled people.

**Table 1:** **Use of adult preventive health services among the physical disability**: **basic characteristics and bivariate analysis**

			**Used**		**Did not use**		**χ** ^**2**^
**Variable name**	**N = 329264**	**%**	**n**_**1**_ **= 53913**	**%**	**n**_**2**_ **= 275351**	**%**	**p-value**
Overall rate of use				16.4		83.6	
Gender							<.001*
Female	137698	41.8	24323	17.7	113375	82.3	
Male	191566	58.2	29590	15.5	161976	84.6	
Age							<.001*
40-44 years	20795	6.3	3224	15.5	17571	84.5	
45-49 years	47257	14.4	8401	17.8	38856	82.2	
50-54 years	44941	13.7	9039	20.1	35902	79.9	
55-59 years	37922	11.5	8516	22.5	29406	77.5	
60-64 years	30296	9.2	7746	25.6	22550	74.4	
65-69 years	35532	10.8	4549	12.8	30983	87.2	
≥70 years	112521	34.2	12438	11.1	100083	89.0	
Educational level							<.001*
Elementary school and under	168180	51.1	27179	16.2	141001	83.8	
Junior high school	49103	14.9	8784	17.9	40319	82.1	
Senior (vocational) high school	45345	13.8	7740	17.1	37605	82.9	
Junior college and university or above	23908	7.3	3491	14.6	20417	85.4	
Unclear	42728	13.0	6719	15.7	36009	84.3	
Marital status							<.001*
Married	192456	58.5	33027	17.2	159429	82.8	
Unmarried	30357	9.2	4630	15.3	25727	84.8	
Divorced or widowed	14209	4.3	2234	15.7	11975	84.3	
Unclear	92242	28.0	14022	15.2	78220	84.8	
Level of urbanization^a^							<.001*
Level one	28966	8.8	3158	10.9	25808	89.1	
Level two	66465	20.2	10372	15.6	56093	84.4	
Level three	47461	14.4	7860	16.6	39601	83.4	
Level four	28536	8.7	4503	15.8	24033	84.2	
Level five	51000	15.5	9053	17.8	41947	82.3	
Level six	39836	12.1	6739	16.9	33097	83.1	
Level seven	43417	13.2	7717	17.8	35700	82.2	
Level eight	23583	7.2	4511	19.1	19072	80.9	
Monthly insured payroll							<.001*
Insured dependents	111998	34.0	14367	12.8	97631	87.2	
<15,840	66219	20.1	10338	15.6	55881	84.4	
16,500-22,800	100385	30.5	18934	18.9	81451	81.1	
24,000-28,800	14864	4.5	3288	22.1	11576	77.9	
30,300-36,300	13942	4.2	3113	22.3	10829	77.7	
38,200-45,800	12768	3.9	2643	20.7	10125	79.3	
48,200-57,800	9088	2.8	1230	13.5	7858	86.5	
Aborigine							<.001*
Yes	7105	2.2	1713	24.1	5392	75.9	
No	322159	97.8	52200	16.2	269959	83.8	
Catastrophic illness/injury							<.001*
Yes	40645	12.3	6072	14.9	34573	85.1	
No	288619	87.7	47841	16.6	240778	83.4	
Chronic diseases							
Cancer							<.001*
Yes	12916	3.9	1695	13.1	11221	86.9	
No	316348	96.1	52218	16.5	264130	83.5	
Endocrine and metabolic disease							<.001*
Yes	136530	41.5	27829	20.4	108701	79.6	
No	192734	58.5	26084	13.5	166650	86.5	
Mental illness							<.001*
Yes	80162	24.4	16410	20.5	63752	79.5	
No	249102	75.7	37503	15.1	211599	84.9	
Disease of the nervous system							<.001*
Yes	72140	21.9	12796	17.7	59344	82.3	
No	257124	78.1	41117	16.0	216007	84.0	
Disease of the circulatory system							<.001*
Yes	184863	56.1	33763	18.3	151100	81.7	
No	144401	43.9	20150	14.0	124251	86.1	
Disease of the respiratory system							<.001*
Yes	81821	24.9	16381	20.0	65440	80.0	
No	247443	75.2	37532	15.2	209911	84.8	
Disease of the digestive system							<.001*
Yes	131055	39.8	27593	21.0	103462	79.0	
No	198209	60.2	26320	13.3	171889	86.7	
Disease of the urinary system							<.001*
Yes	18007	5.5	3325	18.5	14682	81.5	
No	311257	94.5	50588	16.3	260669	83.8	
Disease of the skeletal and muscular system and connective tissue							<.001*
Yes	153030	46.5	30622	20.0	122408	80.0	
No	176234	53.5	23291	13.2	152943	86.8	
Disease of the eyes and auxiliary organs							<.001*
Yes	35599	10.8	6716	18.9	28883	81.1	
No	293665	89.5	47197	16.1	246468	83.9	
Infectious diseases							<.001*
Yes	18318	5.6	3701	20.2	14617	79.8	
No	310946	94.4	50212	16.2	260734	83.9	
Congenital malformation							<.001*
Yes	8459	2.6	1720	20.3	6739	79.7	
No	320805	97.4	52193	16.3	268612	83.7	
Skin and subcutaneous tissue disorders							<.001*
Yes	41891	12.7	8583	20.5	33308	79.5	
No	287373	87.3	45330	15.8	242043	84.2	
Diseases of the blood and blood-forming organs							<.001*
Yes	16524	5.0	3326	20.1	13198	79.9	
No	312740	95.0	50587	16.2	262153	83.8	
Diseases of the ear and mastoid process							<.001*
Yes	27074	8.2	5748	21.2	21326	78.8	
No	302190	91.8	48165	15.9	254025	84.0	
Severity of physical disability							<.001*
Mild	153117	46.5	28742	18.8	124375	81.2	
Moderate	128201	38.9	18860	14.7	109341	85.3	
Severe	44097	13.4	5747	13.0	38350	87.0	
Very severe	3849	1.2	564	14.7	3285	85.4	

^a^Level one: the most urbanized areas.

*P < 0.05.

Under the criteria for assessing model fit, the log-likelihood statistics for the fitted model indicated the model fitted well. This study used statistical software package SAS version 9.3 as an analysis tool. Statistics less than *P* <0.05 were significant.

## Results

### Physically disabled people’s basic information

Table 1 showed that there were 329,264 physically disabled people during 2006 to 2008. Over half of these were male (58.2%; n = 191,566). In the age category, most participants were older than or equivalent to 70 years of age (34.2%; n = 112,521), followed by participants between 45 and 49 (14.4%, n = 47,257). More than half of the participants were married (58.5%; n = 192,456). Participants with education levels less than or equivalent to elementary school accounted for the majority (51.1%; n = 168,180), followed by junior high level (14.9%; n = 49,103). Regarding monthly insured payroll, the insured dependent population accounted for the majority (i.e., children and spouses; 34.0%; n = 111,998). There were few people of aboriginal status, only accounting for 2.2% (n = 7,105). Mildly disabled (46.5%; n = 153,117) was the largest group in the physical disability severity level category.

### Physically disabled people’s free preventive health care utilization

In this study, 16.4% (n = 53,913) of the physically disabled utilized free adult preventive health care (Table 1). Men and women’s utilization rates were 15.5% and 17.7% (*P* <0.05), respectively. The men’s utilization rate was slightly lower than the women’s. Regarding age distribution, the 50 to 64 year-old group had the higher physically disabled utilization rate, more than 20%. The 60 to 64 year-old group had the greatest utilization (25.6%). Married people had a higher utilization rate (17.2%) than did the others (15.2% - 15.7%, *P* <0.05). For education, junior high and senior (vocational) high school had greater utilization rates: 17.9% and 17.1%, respectively. Those with URA Level 8 had the greatest utilization rate (19.1%), and Level 1 had the smallest (10.9%). For income, those with a monthly insured payroll of NT $30,300 to 36,300 (22.3%) had the highest rate. Those with aboriginal status had higher utilization rates (24.1%) than did non-aborigines (16.2%, *P* <0.05). In the relevant chronic diseases category, people with "diseases of the ear and mastoid process" (21. 2%) had a higher utilization rate, and utilization rate for those with cancer (13.1%) was less than those without cancer (16.5%, *P* <0.05). Regarding severity of disability, those with mild disability had the highest utilization rate (18.8%) (*P* <0.05).

### Factors related to use of free adult preventive health care

As shown in Table 2, this study found that gender, age, education, marital status, urbanization of residence area (URA), monthly insured payroll, aboriginal status, catastrophic illnesses status, relevant chronic diseases, and severity of the disability had significant effects on the use of free adult preventive health care by the physically disabled (*P* <0.05).

**Table 2:** **Factors influencing the physical disabled to use adult preventive health services**: **logistic regression analysis**

	**Unadjusted**				**Adjusted**			
**Variable name**	**OR**	**95% CI**		**p-value**	**OR**	**95% CI**		**p-value**
Gender								
Female	1	-	-	-	1	-	-	-
Male	0.85	0.84	0.87	<.001*	0.82	0.80	0.84	0.001*
Age								
40-44 years	1	-	-	-	1	-	-	-
45-49 years	1.18	1.13	1.23	<.001*	1.13	1.08	1.18	<.001*
50-54years	1.37	1.31	1.43	<.001*	1.21	1.16	1.27	<.001*
55-59 years	1.58	1.51	1.65	<.001*	1.25	1.19	1.31	<.001*
60-64 years	1.87	1.79	1.96	<.001*	1.41	1.34	1.48	<.001*
65-69 years	0.80	0.76	0.84	<.001*	0.55	0.52	0.58	<.001*
≥70 years	0.68	0.65	0.71	<.001*	0.46	0.44	0.48	<.001*
Educational level								
Elementary school and under	1	-	-	-	1	-	-	-
Junior high school	1.13	1.10	1.16	<.001*	1.01	0.98	1.04	0.596
Senior (vocational) high school	1.07	1.04	1.10	<.001*	0.99	0.96	1.02	0.519
Junior college and university or above	0.89	0.85	0.92	<.001*	0.95	0.91	0.99	0.026*
Unclear	0.97	0.94	1.00	0.029*	0.99	0.96	1.02	0.609
Marital status								
Married	1	-	-	-	1	-	-	-
Unmarried	1.15	1.11	1.19	<.001*	1.11	1.07	1.15	<.001*
Divorced or widowed	1.04	0.98	1.10	0.200	1.05	0.99	1.11	0.103
Unclear	1.00	0.96	1.03	0.832	0.96	0.92	1.00	0.028*
Level of urbanization^a^								
Level one	1	-	-	-	1	-	-	-
Level two	1.51	1.45	1.58	<.001*	1.55	1.48	1.62	<.001*
Level three	1.62	1.55	1.70	<.001*	1.67	1.60	1.75	<.001*
Level four	1.53	1.46	1.61	<.001*	1.52	1.45	1.60	<.001*
Level five	1.76	1.69	1.84	<.001*	1.79	1.71	1.87	<.001*
Level six	1.66	1.59	1.74	<.001*	1.69	1.61	1.77	<.001*
Level seven	1.77	1.69	1.85	<.001*	1.79	1.71	1.88	<.001*
Level eight	1.93	1.84	2.03	<.001*	1.72	1.63	1.82	<.001*
Monthly insured payroll								
<15,840	1	-	-	-	1	-	-	-
Insured dependents	0.80	0.77	0.82	<.001*	0.92	0.89	0.95	<.001*
16,500-22,800	1.26	1.22	1.29	<.001*	1.19	1.15	1.23	<.001*
24,000-28,800	1.54	1.47	1.60	<.001*	1.26	1.20	1.32	<.001*
30,300-36,300	1.55	1.49	1.63	<.001*	1.27	1.21	1.33	<.001*
38,200-45,800	1.41	1.35	1.48	<.001*	1.17	1.11	1.23	<.001*
48,200-57,800	0.85	0.79	0.90	<.001*	0.84	0.78	0.90	<.001*
Aborigine								
No	1	-	-	-	1	-	-	-
Yes	1.64	1.56	1.74	<.001*	1.21	1.14	1.28	<.001*
Catastrophic illness/injury								
No	1	-	-	-	1	-	-	-
Yes	0.88	0.86	0.91	<.001*	0.86	0.83	0.89	<.001*
Chronic diseases								
Cancer	0.76	0.73	0.81	<.001*	0.87	0.82	0.93	<.001*
Endocrine and metabolic disease	1.64	1.61	1.67	<.001*	1.34	1.31	1.37	<.001*
Mental illness	1.45	1.42	1.48	<.001*	1.18	1.15	1.21	<.001*
Disease of the nervous system	1.13	1.11	1.16	<.001*	0.95	0.93	0.97	<.001*
Disease of the circulatory system	1.38	1.35	1.40	<.001*	1.24	1.21	1.27	<.001*
Disease of the respiratory system	1.40	1.37	1.43	<.001*	1.22	1.19	1.25	<.001*
Disease of the digestive system	1.74	1.71	1.77	<.001*	1.40	1.37	1.43	<.001*
Disease of the urinary system	1.17	1.12	1.21	<.001*	0.97	0.93	1.01	0.124
Disease of the skeletal and muscular system and connective tissue	1.64	1.61	1.67	<.001*	1.29	1.26	1.32	<.001*
Disease of the eyes and auxiliary organs	1.21	1.18	1.25	<.001*	1.02	0.99	1.05	0.200
Infectious diseases	1.32	1.27	1.37	<.001*	1.11	1.06	1.15	<.001*
Congenital malformation	1.31	1.25	1.39	<.001*	1.00	0.95	1.06	0.915
Skin and subcutaneous tissue disorders	1.38	1.34	1.41	<.001*	1.18	1.15	1.22	<.001*
Diseases of the blood and blood-forming organs	1.31	1.26	1.36	<.001*	1.09	1.05	1.14	<.001*
Diseases of the ear and mastoid process	1.42	1.38	1.47	<.001*	1.10	1.07	1.14	<.001*
Severity of physical disability								
Mild	1	-	-	-	1	-	-	-
Moderate	0.75	0.73	0.76	<.001*	0.84	0.82	0.86	<.001*
Severe	0.65	0.63	0.67	<.001*	0.80	0.78	0.83	<.001*
Very severe	0.74	0.68	0.81	<.001*	0.92	0.84	1.01	0.082

^a^Level one: the most urbanized areas.

*P < 0.05.

The logistic regression analysis showed that physically disabled men had a slightly lower free adult preventive health care utilization rate, only 0.82 times that of women (95% CI = 0.80-0 84). In the age aspect, using 40 to 44 year olds as a reference group, the 60 to 64 age group’s utilization rate was 1.41 times greater than the reference rate (OR = 1.41, 95% CI = 1.34-1.48). The lowest rate was among those older than or equivalent to 70 years, which only had 0.46 times that of the 40 to 44 age group (OR = 0.46, 95% CI = 0.44-0.48). There were significant differences between urban and rural life. Utilization by those in Level 8 areas (rural) was 1.72 times higher (OR = 1.72, 95% CI = 1.63-1.82) than by those in Level 1 areas (urban). Using the lowest monthly insured payroll (i.e., less than NT $15,840) as a reference, the NT $30,300 to 36,300 group had 1.27 times the utilization rate of the reference group (OR = 1.27, 95% CI = 1.21-1.33).

Those with aboriginal status had a higher utilization rate, which was 1.21 times higher than that of non-aborigines (OR = 1.21, 95% CI = 1.14-1.28). Those with junior college or university educations or above had a utilization rate of only 0.95 times that of those with elementary school educations and below (OR = 0.95, 95% CI, 0.91-0.99). Those who were married had 1.11 times the utilization rate (OR = 1.11, 95% CI = 1.07-1.15) of those who were unmarried. Those with a catastrophic illness/injury had a lower utilization rate, only 0.86 times (OR = 0.86, 95% CI = 0.83-0.89) that of those without catastrophic illness/injury.

Relevant chronic diseases (except for cancer, diseases of the nervous system, and diseases of the urinary system, which had less utilization than those who were not afflicted), had a higher utilization rate than those not suffering from relevant chronic diseases. Those with “severe” disability had the lowest utilization rate among the severity of disability categories, which was 0.8 times (OR = 0.80, 95% CI = 0.78-0.83) that of the “mild” category.

## Discussion

Comparing Taiwan’s disabled people’s utilization of free adult preventive health care, mental illness was the highest, being 1.47 times that of the physically disabled (OR = 1.47, 95% CI = 1.37-1.45); second was that of the hearing impaired (OR =1.22, 95% CI = 1.19-1.24), which was 1.22 times [16] that of the physically disabled. Compared to the free preventive health care utilization rate of all population (34.2%), the physically disabled had a much lower utilization rate (16.37%) [28].

This study found that women had a higher free preventive health care utilization rate than did men. This was consistent with the results of previous studies [29-31]. It is possible that women are more concerned with health-related messages, and therefore have a higher utilization rate [32]. For age, the group with the lowest utilization rate was the above-70 group. Whether this was related to elderly handicapped persons mostly living in nursing homes and other institutions [33] or because they have geriatric chronic diseases that cause them to frequently visit hospitals or clinics and not necessary for preventive care remains a valuable topic for future research.

For area of residence, the free preventive health care utilization rates among the physically disabled living in URA Levels 5, 6, 7, and 8 were higher than those living in Levels 1, 2, 3, and 4. Preventive health care utilization rates were higher in rural areas than in urban areas. Although urban areas have closer proximity to care, there are no designated personnel to encourage the public to conduct health checks. Rural areas have a slower pace of living, and people like to participate in free preventive health care with their neighbors. In addition, Taiwan’s government has attached importance to the medical gap between urban and rural areas, regularly sending medical patrol vehicles to remote areas to provide medical care and to strengthen publicity. Therefore, those in rural areas have higher preventive health care utilization rates than those in urban areas.

Regarding socioeconomic status, previous studies have shown higher income earners have higher preventive health care utilization rates [34-36]. The study results showed that those with a monthly insured payroll higher than NT $15,840 (except for NT $48,200 to 57,800) have a higher utilization ratio than those earning less than NT 15,840, which was consistent with previous study results. Those with aboriginal status had a higher utilization rate than did non-aborigines. These results implied that since the majority of aboriginal people live in remote areas, the government assigns medical patrol vehicles to regularly provide medical care and preventive health care in remote areas, and then the aborigine’s utilization rate was increased, which reflected the efforts of decreasing the health disparity between residents in urban areas and residents in remote areas. The utilization rate for education level decreased with increases in education. Those with junior college or university or above had the lowest rate. In recent years, various high-end, self-paying preventive health care programs have increased in popularity. Those with junior college or university educations or above and those with monthly insured payroll of NT $48,200 to 57,800 have been selecting these high-end, self-paying preventive health care programs because of their higher social economic statuses.

For marital status, married people had a higher adult preventive health care than did unmarried people. These results support the study conducted by Doescher et al. [29]. Married people tend to have more fixed residence and places of medical care than do unmarried people. They have more opportunities to become familiar with health care professionals and are more likely to accept preventive health care through their recommendations or to be informed by health care messages. In addition, married people have stronger social network (e.g., family members, relatives, and friends) support than do unmarried people, serving as a reminder for their health-promoting behaviors [37,38].

For health status, those with a catastrophic illness/injury had a lower utilization rate than did those who did not have a catastrophic illness/injury. This result differed from that of the American behavioral risk factor surveillance system (BRFSS) [39]. Whether this was due to the physically disabled people’s catastrophic illnesses/injuries making them weak and less mobile is unknown. Regarding the suffering from related chronic diseases category, those with diseases of the digestive system appeared most frequently. This was similar to the results that 58% or more of the patients suffered from constipation, one-third had regular abdominal pain, and 62% had irritable bowel syndrome in a 1998 study on spinal cord injury (SCI) [40]. Regarding the severity of the disability, the greater the level of severity, the lower the probability of preventive health care used. The finding is consistent with that in the study conducted in the United States (2004) [39]. The disabled individuals with higher severity of physical disability would highly depend on assistance of caregivers and leaded to low participation in the preventive health care.

Generally speaking, if the physically disabled people regularly receive free preventive health care, the disease could be early diagnosed and be early treated. If the disease is a minor problem, it could be improved through changes in health behaviors, lifestyle or diet pattern besides necessary medical care received. For the aged people with physical disabilities, if they have chronic diseases, they should visit physicians regularly to treat and control their illnesses. Both regularly receiving preventive health examinations and treating existent diseases are important for the aged and physically disabled people.

### Limitations

In the present study, in order to understand the specific diseases or illness systems influencing the use of preventive health care among the physically disabled people, we included 15 types of chronic diseases as the variable instead of chronic diseases index such as Elixhauser. Thus, this study could not examine the relationship between uses of preventive health care and severity of chronic diseases. In addition, the database did not contain the lifestyle, health beliefs and behaviors of the participants, making further analyses difficult. These were the limitations of this study.

## Conclusions

The study results indicated that the factors that primarily affect physically disabled people’s use of preventive adult health care are gender, age, and education, urbanization of the residence area, income, aboriginal status, catastrophic illness and severity of disability.

There is room for improvement regarding free adult preventive health care for catastrophic illness/injury, among those living in urban areas, unmarried people, those with higher education levels, and seniors who are physically disabled. According to these findings, this study recommends the following: (1) to increase accessibility and convenience, medical institutions should strengthen barrier free space planning. Designated service windows, parking, and bathroom facilities should establish for those who are physically disabled to enhance their mobility. (2) Encourage physicians to actively provide preventive health care services for communities and to take the initiative, reminding handicapped people to obtain regular checkups and follow-ups. (3) Preventive health care for handicapped people requires extra time, labor, and costs than for the public. The government should improve the payment of physicians who conduct preventive health care among handicapped people. Their pay should be increased according to the severity of the patient’s disability to increase the willingness [41] of physicians to perform preventive health care for handicapped people. (4) The extension of media-based publicity and education for the public and medical institutions for the handicapped is recommended. A study in Taipei in 1998 found that 70% of those who had not received adult preventive health care did not know about this service [42].

## Additional file

Additional file 1:
**Range of National Health Insurance Chronic Diseases and ICD-9-code.**

## Abbreviations

BRFSS: Behavioral risk factor surveillance system
CI: Confidence interval
NT$: New Taiwan Dollar
OR: Odds Ratio
SCI: Spinal cord injury
URA: Urbanization level of residence area
