# Supplementary material for: Factors associated with free adult preventive health care utilization among physically disabled people in Taiwan: nationwide population-based study
Source: BMC Health Serv Res. 2014 Dec 5;14:610. doi: 10.1186/s12913-014-0610-5 (PMC4263114; doi:10.1186/s12913-014-0610-5)
Supplement: Additional file 1: — Range of National Health Insurance Chronic Diseases and ICD-9-code. [file 12913_2014_610_MOESM1_ESM.docx]

**Additional file 1: Range of National Health Insurance Chronic Diseases** **and ICD-9-code**

| **No** | **Name of Disease** **& ICD-9-code** |
| --- | --- |
| 1 | **Cancer**  **Cancer**  **ICD-9-code :**  140.x~208.x |
| 2 | **Endocrinopathy and metabolic diseases Dysthyroidism**  **ICD-9-code :**  240.0 240.9 241.0 241.1 241.9 242.00 242.01 242.10 242.11 242.20 242.21 242.30 242.31 242.40 242.41 242.80 242.81 242.90 242.91 243 244.0~244.3 244.8 244.9 245.2~245.4 245.8 245.9 246.0~246.2 246.8 246.9  **Diabetes (01)**  **ICD-9-code :**  250.00~250.03 250.10~250.13 250.20~250 23 250.30~250.33 250.40~250.43 250.50~250.53 250.60~250.63 250.70~250.73 250.80~250.83 250.90~250.93 251.0~ 251.5 251.8 251.9  **Hyperlipemia (19)**  **ICD-9-code :**  272.x  **Wilson's disease (48)**  **ICD-9-code :**  275.1  **Gout (07)**  **ICD-9-code :**  274.0 274.10 274.11 274.19 274.81 274.82 274.89 274.9  **Pemphigus (30)**  **ICD-9-code :**  694.4  **Dermatomyositis (31)**  **ICD-9-code :**  710.3  **Hyperprolactinemia (43)**  **ICD-9-code :**  253.1  **Congenital dysbolism (52)**  **ICD-9-code :**  243 250.01 250.03 250.11 250.13 250.21 250.23 250.31 250.33 250.41 250.43 250.51 250.53 250.61 250.63 250.71 250.73 250.81 250.83 250.91 250.93 253.5 255.2 270 271.0 271.1 272.1 272.6 272.7 272.9 275.1 275.49 275.40 275.41 275.42 277.2 277.5 277.8 277.9  **Dyshormonism induced by adrenal lesion (70)**  **ICD-9-code :**  255.x  **Dyshormonism induced by hypophysis lesion (71)**  **ICD-9-code :**  253.x 254.0 254.1 254.8  **Sexual Precocity (72)**  **ICD-9-code :**  259.0~259.4 259.8 259.9 260  **Parathyroidism (80)**  **ICD-9-code :**  252.0 252.1  **Hypogonadism (93)**  **ICD-9-code :**  256.0 256.1 256.2 256.3 256.4 256.8 256.9 257.0 257.1 257.2 257.8 257.9 258.0 258.1 258.8 258.9 |
| 3 | **Psychiatric diseases Mental illness (47)**  **ICD-9-code :**  290.0 290.10 ~ 290.13 290.20 290.21 290.3 290.40~290.43 290.8 290.9 291.0~291.5 291.81 291.89 291.9 292.0 292.11 292.12 292.2 292.81~292.84 292.89 293.81~293.84 293.89 293.9 294.0 294.10 294.11 294.8 294.9 295.00 295.01~295.05 295.10 ~295.15 295.20~295.25 295.30~295.35 295.50~295.55 295.60~295.65 295.70~295.75 295.80~295.85 295.90 295.900 295.91~295.94 295.940 295.95 296.00 296.000 296.01~296.06 296.10 ~296.16 296.20~296.26 296.30~296.36 296.40~296.46 296.50~296.56 296.60~296.66 296.7 296.70 296.80 296.81 296.82 296.89 296.90 296.900 296.99 297.0~297.3 297.8 297.9 297.90 298.0~298.4 298.8 298.9 298.90 299.00 299.01 299.10 299.11 299.80 299.81 299.90 299.900 299.91 300.00 300.000 300.01 300.02 300.09 300.10 300.100 300.11~300.16 300.9 300.20~ 300.23 300.29 300.3~300.7 300.81 300.82 300.89 301.9 300.90 301.0 301.10~301.13 301.20~301.22 301.3 301.4 301.50 301.51 301.59 301.6 301.7 301.81~301.84 301.89 301.9 301.90 302.0~302.4 302.50~302.53 302.6 302.70~302.76 302.79 302.81~302.85 302.89 302.9 303.90~303.93 304.00~304.03 304.10~304.13 304.20~304.23 304.30~304.33 304.40~304.43 304.50~304.53 304.60 ~304.63 304.70~304.73 307.80 304.81~304.83 304.90~304.93 305.00 ~305.03 305.1 305.20~305.22 305.30~305.33 305.40~305.43 305.50 ~305.53 305.60~305.63 305.70~305.73 305.80 305.81~305.83 305.90~305.93 306.0 ~306.4 306.50~306.53 306.59 306.6~306.9 306.90 307.0 307.1 307.20 307.200 307.21~307.23 307.3 307.40 307.400 307.41~307.50 307.500 307.51~307.54 307.59 307.6 307.7 307.80 307.800 307.81 307.89 307.9 308.0~308.4 308.9 309.0 309.1 309.21~309.24 309.28 309.29 309.3 309.4 309.81~309.83 309.89 309.9 309.90 310.0 ~310.2 310.8 310.9 311 311.0 312.01~312.03 312.10~312.13 312.20 ~312.23 312.30~312.35 312.39 312.4 312.80 312.82 312.89 312.9 312.90 313.0 313.1 313.21~313.23 313.3 313.81 313.82 313.83 313.89 314.00 314.01 314.1 314.2 314.8 314.9 314.90 315.00 315.01 315.02 315.09 315.1 315.2 315.31 315.32 315.39 315.4 315.5 315.8 315.9 315.90 315. 900 316 317 318.0 318.1 318.2 319 |
| 4 | **Neurological diseases Brain tumor complicated with neurotic dysfunction (73)**  **ICD-9-code :**  225.0~225.2 237.5 237.6 239.6  **Parkinson's disease (16)**  **ICD-9-code :**  332.0 332.1  **Amyotrophia (49)**  **ICD-9-code :**  359.2 728.2  **Other central nervous system disorder and hereditary diseases (54)**  **ICD-9-code :**  330.0~330.3 330.8 330.9 331.0~331.4 331.7 331.81 331.89 331.9 333.0 333.1~333.7 333.81~333.84 333.89 333.90 334.0~334.4 334.8 334.9 334.90  **Multiple sclerosis (55)**  **ICD-9-code :**  340  **Infant cerebral palsy and other paralytic syndromes (56)**  **ICD-9-code :**  343.0~343.4 343.8 343.9 343.90 344.00 344.01~344.04 344.09 344.1 344.2 344.30~344.32 344.40~344.42 344.5 344.50 344.81 344.89 344.9  **Epilepsy (15)**  **ICD-9-code :**  345.00 345.01 345.10 345.11 345.2 345.3 345.40 345.41 345.50 345.51 345.60 345.61 345.70 345.71 345.80 345.81 345.90 345.900 345.91  **Myasthenia gravis (51)**  **ICD-9-code :**  358.0  **Distal sensory polyneuropathy (74)**  **ICD-9-code :**  356.4 356.8  **Plexopathy (75)**  **ICD-9-code :**  353.0 353.1 353.8 353.9 353.90  **Trigeminal neuralgia (76)**  **ICD-9-code :**  350.1  **Migraine (77)**  **ICD-9-code :**  346.00 346.01 346.10 346.11 346.20 346.21 346.80 346.81 346.90 346.91  **Spinal cord injury (81)**  **ICD-9-code :**  806.0x~806.3x 806.4 806.5 806.60~806.62 806.69~806.72 806.79 806.8 806.9 907.2 952.0x 952.1x 952.2~952.4 952.8 952.9 |
| 5 | **Circulatory system diseases Heart disease (11)**  **ICD-9-code :**  390 392.x 393 394.x 395.x 396.x 397.x 398.xx 411.0 411.1 411.81 412 413.0 413.1 413.9 414.00~414.05 414.10 414.11 414.19 414.8 414.9 415.11 415.19 416.0 416.1 416.8 416.9 417.0 417.1 417.8 417.9 423.0~423.2 423.8 423.9 424.0~424.3 424.90 424.91 424.99 425.0~425.5 425.7~425.9 426.0 426.10~426.13 426.2~426.4 426.50~426.54 426.6 426.7 426.81 426.89 426.9 427.0~427.2 427.31 427.32 427.41 427.42 427.5 427.60 427.61 427.69 427.81 427.89 427.9 428.0 428.1 428.9 429.0~429.6 429.71 429.79 429.81 429.82 429.89 429.9  **Hypertension (02)**  **ICD-9-code :**  401.0 401.1 401.9 402.00 402.01 402.10 402.11 402.90 402.91 403.00 403.01 403.10 403.11 403.90 403.91 404.00 404.01~404.03 404.10~404.13 404.90~404.93 405.01 405.09 405.11 405.19 405.91 405.99    **Cerebrovascular dysfunction (14)**  **ICD-9-code :**  430 431 432.0 432.1 432.9 433.00 433.01 433.10 433.11 433.20 433.21 433.30 433.31 433.80 433.81 433.90 433.91 434.00 434.01 434.10 434.11 434.90 434.91 435.0~435.3 435.8 435.9 437.0~437.9 437.90 438.0 438.10~438.12 438.19~438.22 438.30~438.32 438.40~438.42 438.50~438.53 438.81 438.82 438.89 438.9  **Atherosclerosis (57)**  **ICD-9-code :**  440.0 440.1 440.20~440.24 440.29~440.32 440.8 440.9 440.90  **Arterial thrombosis (58)**  **ICD-9-code :**  444.0 444.1 444.21 444.22 444.81 444.89 444.9 444.90  **Raynauds disease (26)**  **ICD-9-code :**  443.0  **Kawasaki disease complicated with cardiovascular abnormality (78)**  **ICD-9-code :**  446.1 |
| 6 | **Respiratory system diseases** **Chronic nasal sinusitis (45)**  **ICD-9-code :**  473.0~473.3 473.8 473.9 473.90 473.900 473.901  **Chronic bronchitis (10)**  **ICD-9-code :**  491.0 491.1 491.20 491.21 491.8 491.9 491.90   **Emphysema (20)**  **ICD-9-code :**  492.0 492.8 492.80  **Asthma (06)**  **ICD-9-code :**  493.00 493.01 493.10 493.11 493.20 493.21 493.90 493.900 493.91   **Bronchiectasis (22)**  **ICD-9-code :**  494.0 494.1  **Chronic obstructive pneumonia (21)**  **ICD-9-code :**  496  **Pneumoconiosis (58)**  **ICD-9-code :**  500~505 505.0  **Exopathic pulmonary diseases (60)**  **ICD-9-code :**  506.4 507.0 507.1 507.8 508.0 508.1 508.8 508.9  **Allergic rhinitis (82)**  **ICD-9-code :**  477.x |
| 7 | **Digestive system diseases Digestive ulcer (08)**  **ICD-9-code :**  530.2 531.40 531.41 531.50 531.51 531.60 531.61 531.70 531.71 531.90 531.91 532.40 532.41 532.50 532.51 532.60 532.61 532.70 532.71 532.90 532.91 533.40 533.41 533.50 533.51 533.60 533.61 533.70 533.71 533.90 533.91 534.40 534.41 534.50 534.51 534.60 534.61 534.70 534.71 534.90 534.91 556.x    **Hepatocirrhosis (25)**  **ICD-9-code :**  571.5  **Chronic hepatitis (03)**  **ICD-9-code :**  571.2 571.40 571.41 571.49 571.6 571.8 571.9 572.0~572.4 572.8 576.1  **Functional gastrointestinal disturbance (23)**  **ICD-9-code :**  211.0~211.4 211.9 530.19 535.5 536.8 536.9 555.1 555.2 555.9 564.1 577.1  **Chronic cholecystitis (18)**  **ICD-9-code :**  575.11 |
| 8 | **Urinary system diseases Chronic nephritis (04)**  **ICD-9-code :**  582.0~582.2 582.4 582.81 582.89 582.9 585  **Renal infection (61)**  **ICD-9-code :**  590.00 590.01 590.2 590.80 590.81 590.9 590.90 |
| 9 | **Musculoskeletal system diseases Arthritis (09)**  **ICD-9-code :**  274.0 275.4 696.0 711.0x 714.0~714.3 714.30~714.33 714.4 715.00 715.04 715.09 715.10~715.18 715.20~715.28 715.30 ~715.38 715.80 715.89 715.90 ~715.98 716.0x~716.5x 716.60~716.68 716.8x 716.9x 718.0x 719.0x 720.0 721.3  **Polymyositis (50)**  **ICD-9-code :**  710.4  **Osteoporosis (27)**  **ICD-9-code :**  733.00 733.000 733.001 733.01~733.03 733.09 733.99 733.990  **Lupus erythematosus (24)**  **ICD-9-code :**  695.4 710.0  **Chronic osteomyelitis (95)**  **ICD-9-code :**  730.10~730.18 |
| 10 | **Eye and adnexa diseases**  **Glaucoma (33)**  **ICD-9-code :**  365.000 365.00~ 365.04 365.100 365.10~365.15 365.200 365.20 365.21 365.23 365.24 365.31 365.32 365.41~365.44 365.51 365.52 365.59~365.65 365.81 365.82 365.89 365.9  **Xerophthalmia (34)**  **ICD-9-code :**  375.15  **Retinal degeneration (35)**  **ICD-9-code :**  361.00~361.07 361.10~361.14 361.19 361.2 361.30~361.33 361.81 361.89 362.01 361.02 361.10~362.18 362.21 362.29 362.30~362.37 362.40~362.43 362.60~362.66 362.70~362.77 362.81~362.89 362.9  **Macular degeneration (36)**  **ICD-9-code :**  362.50~362.56  **Uveitis (37)**  **ICD-9-code :**  360.11 360.12  **Vitreous hemorrhage (38)**  **ICD-9-code :**  379.23  **Corneal degeneration (39)**  **ICD-9-code :**  371.40~371.46 371.48 371.49 |
| 11 | **Infectious diseases Tuberculosis (17)**  **ICD-9-code :**  010.00~010.06 010.10~010.16 010.80~010.86 010.90~010.96 011.00~011.06 011.10~011.16 011.20~011.26 011.30~011.36 011.40~011.46 011.50~011.56 011.60~011.66 011.70~011.76 011.80~011.86 011.900 011.90~011.96 012.00~012.06 012.10~012.16 012.20~012.26 012.30~012.36 012.800 012.80~012.86 013.00~013.06 013.10~013.16 013.20~013.26 013.30~013.36 013.40~013.46 013.50~013.56 013.60~013.66 013.80~013.86 013.900 013.90~013.96  014.00~014.06 014.800 014.80~014.86 015.00~015.06 015.10~015.16 015.20~015.26 015.50~015.56 015.60~015.66 015.70~015.76 015.80~015.86 015.900 015.90~015.96 016.00~016.06 016.10~016.16 016.20~016.26 016.30~016.36 016.40~016.46 016.50~016.56 016.60~016.66 016.70~016.76 016.900 016.90~016.96 017.00~017.06 017.10~017.16 017.20~017.26 017.30~017.36 017.40~017.46 017.50~017.56 017.60~017.66 017.70~017.76 017.80~017.86 017.90~017.96 018.80~018.86 018.900 018.90~018.96  **Onychomycosis (29)**  **ICD-9-code :**  110.1 |
| 12 | **Congenital malformation Congenital malformation disease (62)**  **ICD-9-code :**  740.0~740.2 741.00~741.03 741.900 741.90~741.93 742.0~742.4 742.51 742.53 742.59 742.8 742.9 742.90 743.00 743.03 743.06  743.10 ~743.12 743.200 743.20~743.22 743.30~743.37 743.39 743.4x~743.5x 743.61~743.66 743.69 743.8 743.9 744.00~744.05 744.09 744.1 744.21~744.24 744.29 744.3 744.41~744.43 744.46 744.47 744.49 744.5 744.81~744.84 744.89 744.9 745.0 745.10~745.12 745.19 745.2~745.5 745.60 745.61 745.69 745.7~ 745.9 746.00~746.02 746.09 746.1~746.7 746.81~746.87 746.89 745.9 746.90 747.0 747.10 747.11 747.20~747.22 747.29 747.3 747.40~747.42 747.49 747.5 747.60~747.64 747.69 747.81 747.82 747.89 747.9 748.0~748.5 748.60 748.61 748.69 748.8 748. 9 749.00~749.04 749.10~749.14 749.20~749.25 750.0 750.10~750.16 750.19 750.21~750.27 750.29 750.3~750.9 751.0~751.5 751.60~751.62 751.69 751.7~751.9 751.90 752. 0 752.10 752.11 752.19 752.2 752.3 752.40~ 752.42 752.49 752.51 752.52 752.61~752.65 752.69 752.7~752.9 753.0 753.10~753.17 753.19~753.23 753.29 753.3~753.9 754.0~754.2 754.30~754.33 754.35 754.40~754.44 754.50~754.53 754.59~754.62 754.69~754.71 754.79 754.81 754.82 754.89 755.00~755.02 755.10~755.14 755.20~755.39 755.4 755.50~755.67 755.69 755.8 755.9 756. 0 756.10~756.17 756.19 756.2~756.4 756.50~756.56 756.59 756.6 756.70 756.71 756.79 756.81~756.83 756.89 756.9 756.90 757.0 757.2 757.31~757.33 757.4~757.6 757.8 757.9 758.0~758.7 758.81 758.89 758.9 759.0~759.7 759.81~759.83 759.89 759.9 759.90 |
| 13 | **Skin and subcutaneous tissue diseases Dry tinea (28)**  **ICD-9-code :**  696.1 696.10 696.2~696.5 696.8  **Systemic eczema (32)**  **ICD-9-code :**  692.0~692.6 692.70 692.71 692.73~692.75 692.79 692.81~692.83 692.89 692.9 692.90  Lyme disease (bird's leg disease) **(79)**  **ICD-9-code :**  985.1  **Leukasmus (83)**  **ICD-9-code :**  709.01  **Sebacous dermatitis (84)**  **ICD-9-code :**  690.10~690.12 690.18 690.8  **Amyloid deposits (focus area> 30% of the whole body surface area) (85)**  **ICD-9-code :**  277.3  **Pemphigoid (86)**  **ICD-9-code :**  694.4 694.5 694.60 694.61  **Dermatitis herpetiformis (87)**  **ICD-9-code :**  694.0~694.3  **Familial benign chronic pemphigus (88)**  **ICD-9-code :**  694.60 694.61  **Epidermolysis Bullosa (89)**  **ICD-9-code :**  757.39 757.390  **Serious ichthyosis (including lamellar ichthyosis and ichthyosiform erythroderma) (90)**  **ICD-9-code :**  757.1  **Follicular keratosis (91)**  **ICD-9-code :**  757.2 757.3 757.31  **Progressive systemic sclerosis (92)**  **ICD-9-code :**  710.1  **Chronic Urticaria (98)**  **ICD-9-code :**  708  **Atopic dermatitis (99)**  **ICD-9-code :**  691.8 |
| 14 | **Blood and hematopoietic organ diseases Chronic anemia (40)**  **ICD-9-code :**  280.0 280.1 280.8 280.9 281.0~281.4 281.8 282.0~282.4 282.6 282.61~282.63 282.69 282.7 282.8 283.0 283.10 283.11 283.19 283.2 283.9 284.0 284.8 285.0 285.21 285.22 285.29 285.8 285.9  **Purpura (41)**  **ICD-9-code :**  287.0~287.5 287.8 287.9  **Haemophilia (63)**  **ICD-9-code :**  286.0~286.7 286.9  **Myelodysplastic Syndrome (MDS) (96)**  **ICD-9-code :**  238.75  **Refractory anemia**  **ICD-9-code :**  285.0  **RARS**  **ICD-9-code :**  238.72  **CMMOL**  **ICD-9-code :**  205.1  **RAEB**  **ICD-9-code :**  238.73  **RAEB-t**  **ICD-9-code :**  238.7  **Primary thrombocythaemia (97)**  **ICD-9-code :**  238.71 |
| 15 | **Ear and papillary diseases**  **Chronic tympanitis (46)**  **ICD-9-code :**  381.1 381.10 381.19 381.2 381.20 381.29 381.3~381.5 381.52  381.6 381.61~381.63 381.7 381.8 381.81 381.9 382.1~382.3  **Vestibulum auris internae pathological changes (44)**  **ICD-9-code :**  386.0 386.01~386.04 386.1 386.10 386.12 386.2 386.3 386.31~386.35 386.4 386.41~386.43 386.48 386.5 386.51 386.53 386.55 386.8 386.9  **Sensorineural Tinnitus (100)**  **ICD-9-code :**  388.3 |
| 16 | **Others**  **Follow-up medication after organ transplantation (13)**  **ICD-9-code :**  V42.0~V42.7 V42.81~V42.84 V42.89 V42.9  **Leprosy(Hansen's Disease) (64)**  **ICD-9-code :**  030.0~030.3 030.8 030.9 030.90  **Hemorrhoids (65)**  **ICD-9-code :**  455.x  **Prostatic hypertrophy (66)**  **ICD-9-code :**  600.0~600.3 600.9  **Endometriosis (42)**  **ICD-9-code :**  617.x 617.90  **Menolipsis syndrome (67)**  **ICD-9-code :**  627.0~627.4 627.8 627.9  **Urinary incontinence (68)**  **ICD-9-code :**  625.6 788.30~788.34 788.39  **Yusho (polychlorinated biphenyl poisoning) (69)**  **ICD-9-code :**  982.0 989.40  **Congenital immunodeficiency disorders (53)**  **ICD-9-code :**  279.00~279.06 279.09 279.10 279.11 279.13 279.2 279.3 279.30 279.4 279.8 279.9   **Chronic prostatitis (proved by the sampled prostate secretion) (94)**  **ICD-9-code :**  601.1 601.90 |
